# Reports on the Hospitals of the United Kingdom

**Published:** 1910-02-19

**Authors:** Henry Burdett


### REPORTS
### ON THE
### Hospitals of the United Kingdom.

By SIR HENRY BURDETT, K.C.B., K.C.V.O.

### XXVII.—TUNBRIDGE WELLS HOSPITALS.

### THE GENERAL AND THE HOMŒOPATHIC.

There has been some difference of opinion and much debate in the past as to the relative merits and efficiency of the General Hospital and the Homœopathic Hospital for General Diseases at Tunbridge Wells. We thought it essential, therefore, to inspect both and judge them on their merits. We found in practice that no comparison could be fairly drawn, and that much of the pother must have been due to the struggle for existence on the part of one or both. The Homœopathic Hospital is a little 14-bedded institution with a small out-patient department located in an old house, which has been added to, and where a mortuary has been erected. The General Hospital to-day is a modern, up-to-date building, on which from £20,000 to £30,000 have recently been spent, and where the work of reconstruction has, on the whole, been well done. It contains 66 beds, and has one of the most attractive and beautiful children's wards imaginable, which is perfectly kept. There is also a large out-patient department, recently built; but, despite this, the arrangements for the casualty department are defective, for it has no small theatre and no recovery room accommodation, though adenoids and other operation cases are dealt with in considerable numbers. Where so much has been done we are certain that attention has only to be called to the matter to ensure that steps will be taken to make the casualty department complete and up to date. But, apart from this, it will be seen that the two hospitals mentioned have nothing in common for purposes of comparison, and the supporters of each would be well advised to discountenance controversy for the future and to stick to their own proper business.

### THE GENERAL HOSPITAL.

This Hospital was established in 1828, and rebuilt mainly in 1905. It contains 66 beds, the average occupancy being 57. In 1902, in a speech at Tunbridge Wells, we appealed to all classes to reconstruct the old buildings and to provide a modern hospital. This course has been followed. Our inspection of the new hospital in October 1909 was looked forward to with pleasurable anticipation. We rejoice to be able heartily to congratulate all who have had a hand in providing the present modern, up-to-date hospital.

In dealing with this hospital we must first treat it as a modern hospital, recently planned by Mr. Percy Adams, the architect, who has evidently taken pains and done his best. The wards are all good; two wards of 24 beds each are rather too large, as we hold, but they are good in the sense of proportion, window area, ventilation, heating, and so forth. The bathrooms, too, are good; but the ward sculleries are much too small for the size of the wards, and although the details are well thought out they must render the work of the staff unnecessarily arduous and difficult for want of space to turn round in. The ward unit fails, too, in the sense that no rooms have been provided for (1) clinical work, which has now to be done in the wards; (2) patients' clothes; and (3) a larder or food room. The provision for the baths, abutting on the ward kitchens, now used, though well ventilated, is not ideal. The same may be said of the operation theatre unit, for we did not expect to find in so recent a building that the only provision for washing up, and cleaning instruments, etc., after an operation, are the sinks in the theatre itself. This unit is not ideal, either, and might be greatly improved. The children's ward is good in every way, and cannot fail to bring popularity to the hospital.

#### General Condition.

The general condition of the hospital throughout is good. The wards are attractive and well kept, and where everything is maintained at so high a level of efficiency it was remarkably interesting to note in so new a building that one sister could attain a height of efficiency which gave her ward and its offices a specially smart appearance, which could be felt and noticed by an experienced observer. As a general detail we may express surprise that, instead of teak floors, the whole of those in the new wards are covered with linoleum. This is very unusual, if not unique, and it must mean a large additional outlay later on, and be a source of increasing anxiety to the staff and the management. We wonder how it came about that so doubtful an experiment should have been sanctioned. The baths are all porcelain of the very best make and pattern, free from all wooden and other fittings, and we congratulate the Committee on their courage in selecting them, for they should wear for many years if properly taken care of, and so prove a valuable asset and a real economy as time passes. We have found Rufford baths of this material in a large hospital in good condition after forty years' wear.

The kitchen shows economical working, and the matron has the domestic and housekeeping departments well under her control. The tea, though costing the same—1s. 3d. per lb.—was infinitely better quality here than that at the Sussex County

Hospital. The bread, however, struck us as being imperfectly baked, and might be better. Some more and better planned fittings in the linen room and ventilation in the linen cupboards throughout the hospital are called for. The old buildings have been utilised to provide accommodation for the resident and nursing staff. The conversion on the whole has been creditably done, but the cubicles for some of the nurses we should like to see done away with. The fact that what was practically the whole of the old buildings of the hospital should have been found necessary to provide this accommodation is one more proof of the greatly increased cost of maintaining a modern hospital compared with one of the last generation. This modern, up-to-date hospital shows the difference in the quality of the work done by women as compared with that of men. Here the former is highly creditable to everybody concerned, whilst the porters' work in the mortuary and elsewhere is neglected or done in a slovenly way, and needs much closer supervision. It would appear as if the notable cleanliness to be met with everywhere else had not made the slightest impression upon the porters or improved their relative inefficiency as workers.

### Mortuary and Post-mortem Department.

There is a very tasteful and simple mortuary chapel, which does credit to those who thought it out and planned it. The post-mortem room has no hot-water supply, but only an old gas fire, which should not be. The cleanliness of this place has yet to be achieved, and the condition of the cupboards and other things is insanitary and discreditable. The whole place wants turning out, thoroughly cleansing, and keeping in order.

### New Out-patient Department.

This consists of a waiting hall, with physicians' and surgeons' consulting-rooms and annexes. As we have already said, the casualty department as at present constituted fails to meet the conditions of work it has to fulfil, and so should be extended and reconstructed without delay. We were unable to judge definitely without a plan of the site, but this department struck us as exhibiting an absence of thought and knowledge in planning which is rather surprising in a modern building of this kind. There was an empty room full of rubbish of sorts, and the end of the out-patient hall contained empty cases and hampers, all of which were unsightly and ought not to be tolerated. The same absence of supervision, or careful oversight and provision, has permitted the post-mortem room to be used as a store, or something like it. These are relatively small matters; but they are apt to grow, and the male staff should be quickened into activity and have esprit driven into them, for their work at present is not what it might and ought to be. It does an injustice, too, to the high efficiency of the other departments.

### THE HOMŒOPATHIC HOSPITAL.

This small Hospital is very well kept; all the floors are covered with oilcloth, and the wards are airy, bright, and attractive. The furniture, too, is well chosen, and the screens are dainty also. The hospital gave us the impression of being conducted upon lines which must make it popular with the patients, who, we expect, may grow to regard it as a home after a short residence. If there is a demand for homœopathic treatment in Tunbridge Wells this little institution should meet it adequately and well. All the patients were happy and comfortable, and evidently considered themselves fortunate to be there in the day of sickness. There are two committees—one of men and one of women. That the latter do not always look after and provide for the domestic side of hospital work is proved by the fact that the furniture of the nurses' and servants' rooms is not sufficient, there being an absence of wash-hand-stands, chairs, etc. The matron has been here for fifteen years; the present excellent condition of the hospital shows that she does her work well, and the committee might wake up and provide her with a little more money for the supply of necessaries of the kind we have just mentioned. The mortuary, as we have said, though simple, is adequate.

The **Chester General Infirmary** will be reported on next week.

